# Connecting lattice and molecular vibrations to organic crystal properties

**DOI:** 10.1107/S2052252524012326

**Published:** 2025-01-01

**Authors:** Luca Catalano

**Affiliations:** a Dynamic Molecular Materials Laboratory, Department of Life Sciences University of Modena and Reggio Emilia Modena 41125 Italy; b Department of Chemistry University of Rochester Rochester NY 14627 USA

**Keywords:** lattice vibrations, molecular vibrations, lattice dynamics, quantum crystallography, anharmonicity, molecular crystals

## Abstract

Understanding dynamic processes in molecular crystals is becoming crucial for the development of next-generation smart crystalline materials. In this context, Zwolenik & Makal [(2025). *IUCrJ*, **12**, 23–35] shed light on the complex dynamics–structure–properties relation of a pyrene derivative by correlating molecular and lattice anharmonic vibrations with the unusual thermal expansion of the compound.

Molecular crystals are traditionally considered static, brittle, and inert. This view of crystalline materials has deep roots in the chemistry community, as exemplified by the (in)famous statement of Nobel prizewinner Leopold Ruzicka that ‘crystals are a chemical graveyard’. Over the decades, giants of the organic solid-state community have challenged this concept with seminal contributions such as the study of the photoreactivity of cinnamic acid and its derivatives from Schmidt (1971 ▸), the work of Peggy Etter on macroscopic dynamics of metal complexes (Etter & Siedle, 1983 ▸), and the fundamental studies of lattice dynamics of Dunitz & Bürgi (Bürgi & Dunitz, 1983 ▸; Bürgi & Capelli, 2000 ▸), to cite just a few examples.

Building upon these pioneering works, there has been a blossoming of studies in the last two decades shedding light on the complex dynamic landscape of crystalline materials from molecular-scale motion, at the core of the growing field of solid-state molecular machines (Vogelsberg & Garcia-Garibay, 2012 ▸), to macroscopic dynamic processes, such as crystal jumping, shape shifting, elastically and plastically deforming, and self-healing, induced by external stimuli (light, heat, pressure, mechanical stress, electric and magnetic fields), opening new perspectives in the design and synthesis of innovative smart crystalline materials (Awad *et al.*, 2023 ▸). The understanding of dynamic phenomena has progressed alongside the advancement of experimental techniques and computational methods capable of characterizing these often-overlooked effects, for example, solid-state NMR, variable-temperature, pressure-dependent and time-resolved diffraction experiments, UV–visible spectroscopy, different vibrational spectroscopy techniques (IR, Raman and THz), and enhanced sampling molecular dynamics.

In this context, an in-depth characterization of the thermal fluctuations of atomic nuclei, manifesting as molecular and lattice vibrations, is becoming essential to correlate dynamics, structures and functions of crystalline systems. The vibrations in organic crystals based on light elements, generally described within the harmonic approximation, are largely characterized by strong anharmonic behavior especially at ambient conditions; this comes with inevitable disruptive effects on the electronic, optical, mechanical and thermal properties, solid-state reactivity, and polymorphism (Schweicher *et al.*, 2019 ▸; Asher *et al.*, 2020 ▸; Aree *et al.*, 2022 ▸; Ascher *et al.*, 2023 ▸; Catalano *et al.*, 2024 ▸). Low-frequency Raman spectroscopy, IR spectroscopy, THz time-domain spectroscopy, neutron and X-ray diffraction are the elective experimental tools for obtaining information on anharmonic vibrations at variable pressure and temperature. In particular, X-ray diffraction combined with quantum mechanical calculations, namely quantum crystallography, is becoming a powerful method for obtaining information on lattice and molecular vibrations through the anisotropic refinement of displacement parameters, accessing precious structural information on the dynamics and anharmonicity of crystalline materials (Bürgi *et al.*, 2000 ▸; Grabowsky *et al.*, 2017 ▸).

In this issue of **IUCrJ**, Zwolenik and Makal report a comprehensive investigation into the anharmonic effects in the crystal structure of a polymorph (the β form) of 1,3-di­acetyl­pyrene, a luminescent material previously reported by the authors (Zwolenik *et al.*, 2024 ▸), trying to correlate molecular and lattice vibrations with the interesting thermal properties of the system (Fig. 1 ▸), namely a remarkable negative thermal expansion (NTE) with a linear thermal expansion coefficient of −199 (6) MK^−1^ at room temperature (Zwolenik & Makal, 2025 ▸).

A major finding of the study is the quantification of anharmonicity in the molecular vibrations of this crystalline material within the Gram–Charlier formalism. The authors successfully refined the atomic displacements using Hirshfeld atom refinement, providing a deeper insight into the role of anharmonic oscillations that contribute to the observed NTE. The ability to detect and model these subtle anharmonic effects in an organic material, composed solely of light elements, over a broad range of temperatures (90–390 K) and with in-house equipment, is a notable achievement, especially since previous studies have generally focused on heavier elements, cryogenic temperatures, and diffraction experiments based on synchrotron light sources.

The study highlights that the anomalous thermal expansion behavior in a specific crystallographic direction is linked to the transverse vibrations of oxygen atoms in the acetyl groups of the pyrene derivative. These vibrations, coupled with the rigidity of the C—H⋯O hydrogen bonds of the structure, lead to an expansion in one direction and contraction in another, contributing to the overall colossal NTE coefficient, confirming the prominent role of anharmonicity in dictating thermal expansion in molecular crystals (Juneja *et al.*, 2024 ▸).

This work exemplifies the application of modern quantum crystallography tools to study dynamic effects in organic materials. The refinement of anharmonic oscillations, even at moderate resolution, high temperature and with the use of in-house facilities, marks a significant advance in crystallographic methodology. This approach could prove beneficial for the whole organic solid-state community, especially when other experimental techniques are not applicable and/or available, as in this work. Furthermore, the authors emphasize the potential of these methods to broaden the scope of anharmonic studies, enabling detailed investigations of more complex organic systems that were previously considered challenging due to limitations in scattering power.

In conclusion, the study provides an in-depth analysis of the anharmonic behavior of the β form of 1,3-di­acetyl­pyrene, demonstrating the power of combining experimental X-ray diffraction with sophisticated computational approaches. The results not only deepen our understanding of NTE but also pave the way for further studies on the dynamic properties of organic crystalline materials, offering new avenues for designing materials with tailored properties.

## Figures and Tables

**Figure 1:**
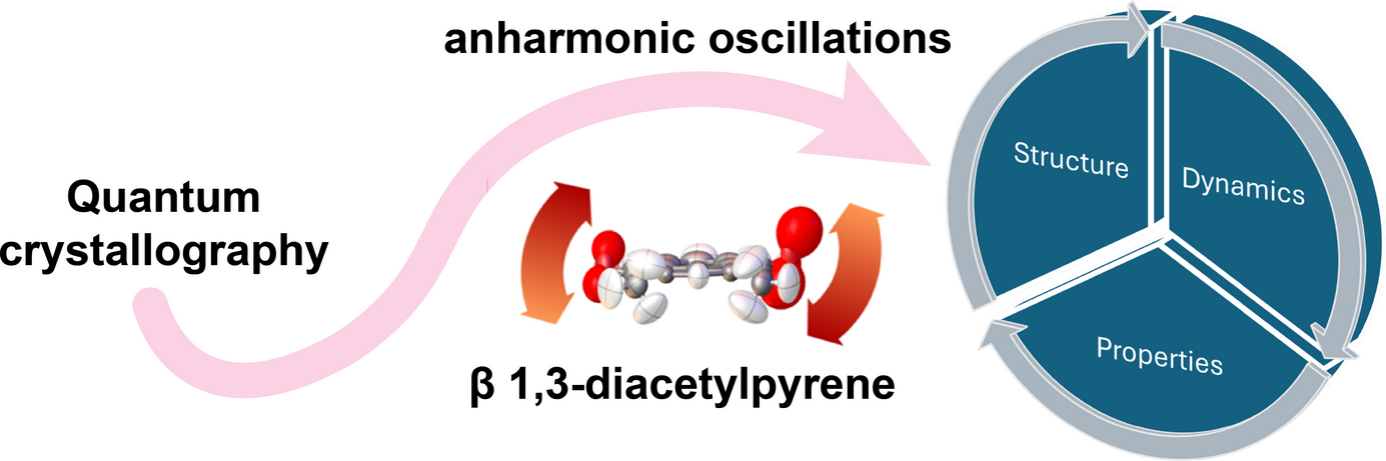
The strategy devised by Zwolenik and Makal to shed light on the structure, dynamics and properties relations of the β form of 1,3-di­acetyl­pyrene (Zwolenik & Makal, 2025 ▸).
